# Ethnomedicinal, Phytochemical and Pharmacological Investigations of *Tetradenia riparia* (Hochst.) Codd (Lamiaceae)

**DOI:** 10.3389/fphar.2022.896078

**Published:** 2022-06-02

**Authors:** Sujogya Kumar Panda, Zilda Cristiani Gazim, Shasank S. Swain, Marisa Cassia Vieira de Araujo Bento, Jéssica da Silva Sena, Marie Jeanne Mukazayire, Luc Van Puyvelde, Walter Luyten

**Affiliations:** ^1^ Department of Biology, Animal Physiology and Neurobiology Section, KU Leuven, Leuven, Belgium; ^2^ Centre of Environment Climate Change and Public Health, RUSA, Utkal University, Bhubaneswar, India; ^3^ Chemistry Laboratory of Natural Products, Graduate Program in Animal Science and Biotechnology Applied to Agriculture, Paranaense University, Umuarama, Brazil; ^4^ Division of Microbiology and NCDs, ICMR-Regional Medical Research Centre, Bhubaneswar, India; ^5^ Department of Pharmacy, School of Pharmacy and Medicine, College of Medicine and Health Sciences, University of Rwanda, Huye, Rwanda

**Keywords:** ethnopharmacology, traditional folk medicine, diterpenes, 8(14),15sandaracopimaradiene-7 α, 18-diol, 14-hydroxy-9-epi(E)-caryophyllene, 6,7-dehydroroileanone, computational analysis

## Abstract

*Tetradenia riparia* Hochsteter codd. (*Lamiaceae*) in its native African continent, is considered one of the most popular aromatic medicinal plants. In folk medicine it may be used as an infusion to treat respiratory problems, cough, headache, stomach pain, diarrhea, fever, malaria, and dengue; and in the form of compresses it is applied for the relief of headaches and toothaches. The species *T. riparia* has been researched for decades to isolate and identify chemical constituents present in extracts or essential oil obtained from the leaves, floral buds, or stems of this plant. The present study reviews the scientific literature on ethnomedicinal, phytochemical, and pharmacological aspects of *T. riparia*. We discuss issues related to the botanical and geographical description of the species, ethnobotanical uses, phytochemical studies on its essential oil and extracts, and biological activities of *T. riparia.* Several compounds have already been isolated from leaves, such as ibozol, 7α-hydroxyroileanone, 1′,2′-dideacetylboronolide, 8(14),15-sandaracopimaradiene-7α,18-diol; 5,6-dihydro-α-pyrone and α-pyrone. Terpenes predominated in the essential oil, comprising monoterpenes, sesquiterpenes, diterpenes, hydrocarbons, and oxygenates. Most phytocompounds were isolated from the leaves and flower buds, namely fenchone, 14-hydroxy-9-epi (E)-caryophyllene, 9β, 13β-epoxy-7-abietene, and 6,7-dehydroroileanone. These compounds provide the species a high pharmacological potential, with antimicrobial, antioxidant, antitumor, analgesic, anti-leishmania, anti-tuberculosis, and anti-parasitic activities. Therefore, this species is a promising herbal medicine.

## 1 Introduction

The species *T. riparia* is native to the African continent, where is considered one of the most popular aromatic medicinal plants. It is usually planted close to homes to ward off mosquitoes, illustrating the repellent potential of its essential oil (Van Puyvelde et al., 1986). In folk medicine, its leaves are used to treat several diseases such as malaria, angina, yaws, helminths, dental abscesses, gastroenteritis, several types of fevers, headaches and other pains. Its leaves are also used for the conservation of foodstuffs in traditional silos (Van Puyvelde et al., 1986).

The essential oil produced from its leaves and flower buds has an orange color and consists mostly of terpenoid compounds such as monoterpenes, sesquiterpenes, and diterpenes (in each case as hydrocarbons or oxygenated), whose concentration varies with the seasons (Gazim et al., 2010; Zardeto-Sabec et al., 2020).

Studies with *T. riparia* have demonstrated many biological activities, such as antimicrobial (Gazim et al., 2010; York et al., 2012; Elaka et al., 2020; Scanavacca et al., In Press), antioxidant (Fernandez et al., 2017), antitumor (Gazim et al., 2014), analgesic (Gazim et al., 2010), anti-leishmania (Cardoso et al., 2015; Demarchi et al., 2015; Leitão et al., 2020), antituberculosis (Baldin et al., 2018), antiparasitic (de Melo et al., 2015), as well as acaricidal and larvicidal (Lorenzi and Matos, 2008; Gazim et al., 2011; Fernandez et al., 2014; Zardeto-Sabec et al., 2020).

Notwithstanding extensive traditional reports on the use of this plant, little progress was made on its bioactivity during 1990–2015. Its phytochemistry was extensively described during 1980–1990, but scientific evidence for its use in various diseases is scarce, both *in vitro* and *in vivo*. Thus, the present review aims to summarize the ethnomedicinal, phytochemical and pharmacological aspects of *T. riparia*. It covers the ethnobotany, chemo-profiling, and biological evaluation (*in vitro* and *in vivo*) of *T. riparia* extracts and essential oils, as well as compounds therein, with a critical discussion of their toxicity, structure activity relationship, computational investigation, as well as suggestions for further basic and clinical research.

## 2 Botanical Description, Geographic Distribution

**FIGURE 1:**
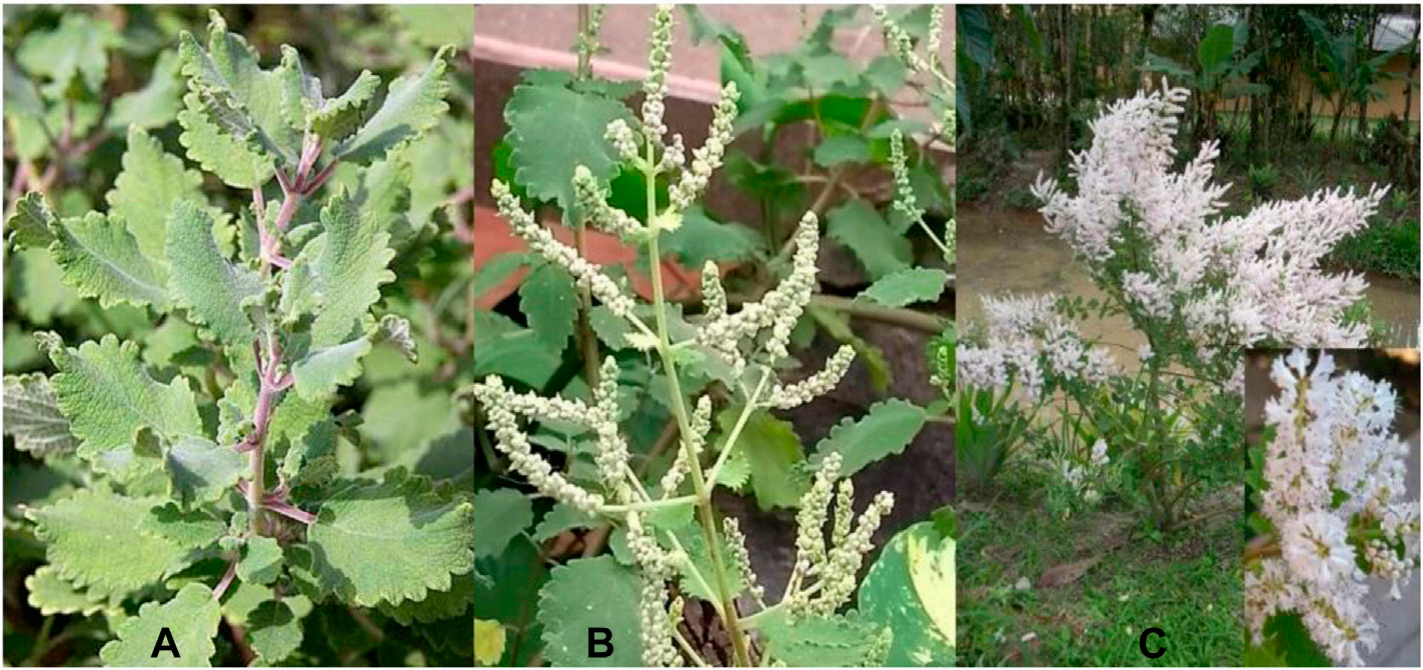
*T. riparia* culture planted in the medicinal garden of Paranaense University. Umuarama, Parana, Brazil. **(A)**: leaves **(B)**: Flower buds **(C)** Open flowers. Source: the authors.

Species of the genus *Tetradenia* are generally aromatic shrubs 1–3 m high, dioecious, soft and very branched. The stems are brittle, semisucculent, semi-juicy, aromatic, rather stout, at first 4-angled and glandular-pubescent, becoming terete and glabrous with age; the bark is pale brown. The leaves are petiolate, ovate-oblong to round, with glandular trichomes distributed on both surfaces (Codd, 1983, Phillipson and Steyn 2008), as can be seen in Figure 1A. The epidermis is uniseriate, with irregular cells, the cells on the adaxial surface being larger than the epidermal cells on the abaxial surface, surrounded by a cuticle and containing glandular (capitate and peltate) and non-glandular trichomes (Martins et al., 2008). Flowering occurs only in subtropical, temperate and frost-free areas (Blythe et al., 2020). The flower buds (Figure 1B) begin to appear in winter (June in the Southern hemisphere), with opening of the flowers in July. The inflorescences appear in large, branched terminal panicles (Figure 1C) that are white to pale mauve in color (Zardeto-Sabec et al., 2020). *T. riparia* has eight synonyms: *Basilicum myriostachyum* (Benth.) Kuntze, *Basilicum riparium* (Hochst.) Kuntze, *Gumira ferruginea* (A.Rich.) Kuntze, *Iboza riparia* (Hochst.) N.E.Br., *Moschosma myriostachyum* Benth., *Moschosma riparium* Hochst., *Plectranthus riparius* Hochst., *Premna ferrugínea* A. Rich. (Powo, 2022).

**FIGURE 2:**
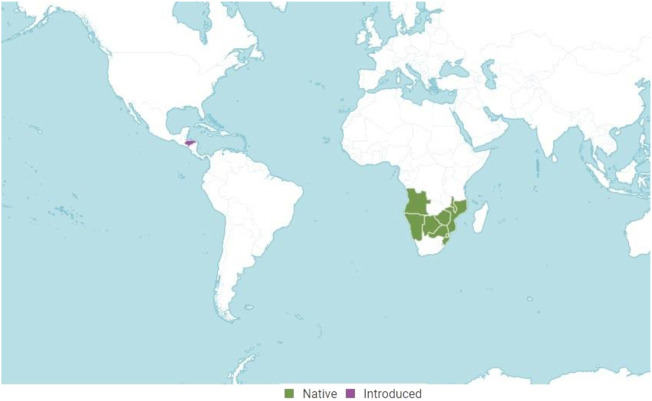
Geographical distribution of *T. riparia* (Source: Plants of the World Online, Royal Botanic Gardens, Kew. 2022. Licensed under Creative Commons Attribution CC BY).

In South Africa, where it is native, *T. riparia* is one of the most popular aromatic medicinal plants and is usually planted close to homes to repel mosquitoes (Van Puyvelde et al., 1986 and 1987; Weaver et al., 1994; Campbell et al., 1997; Van Puyvelde and de Kimpe, 1998). The typical range of *T. riparia* is at lower elevations, from near sea-level, or in more humid habitats (Phillipson and Steyn, 2008), such as along river banks, forest margins, dry wooded valleys and hillsides (Njau and Ndakidemi, 2017). However, it is well adapted in tropical regions such as Brazil. In this country, it is found in more humid areas such as the northern region (Godoy et al., 1999), but also in regions with lower humidity, such as the southern region (Gazim et al., 2010). The vegetative propagation of the *T. riparia* species occurs by cuttings of woody material, coming from the same matrix, with the objective of maintaining in the seedlings the genetic characteristics of the matrix plants, such as uniformity and precocity in production (Pinheiro et al., 2021). In Brazil, the population does not use it as a medicinal plant, but as an ornamental; thus, it is found in parks and gardens (Zelnik et al., 1978). The natural distribution ranges from South Africa to Angola, Botswana, KwaZulu-Natal, Malawi, Mozambique, Namibia, Northern Provinces, Swaziland, and Zimbabwe. It was introduced in Honduras (Figure 2), from where it was probably distributed throughout the American continent (Powo, 2022).

## 3 Ethnobotanical Uses

The medicinal value of *T. riparia,* popularly known as umuaravumba, is well known among the native people of Rwanda, and, accordingly, several medicinal applications have been reported in Rwanda but also elsewhere in eastern Africa (Van Puyvelde et al., 1981). Rwandese people often cultivate the plant around their houses, and use it as a remedy against a wide range of diseases including malaria, angina, yaws, helminthic diseases, gastroenteritis, gonorrhea, diarrhea, dental abscesses, headache, and several kinds of fevers and aches (Van Puyvelde et al., 1986). There are also reports about its use to treat toothaches and diseases caused by worms, bacteria, or fungi (de Melo et al., 2015). In addition to being used as a medicinal plant, the leaves are also used to conserve food in traditional silos, as well as in the dry storage of crops, in order to repel insects (Van Puyvelde et al., 1986). The application in the conservation of grains during storage in Rwanda was validated by Weaver et al. (1994), using the essential oil extracted from the leaves on *Zabrotes subfasciatus*, a beetle that infests the bean grains (see item 5.6 Insecticidal activity on *Zabrotes subfasciatus*)*.*

According to Lorenzi and Matos (2008), *T. riparia* is traditionally used as an infusion, for the treatment of respiratory problems, cough, headache, stomach pain, diarrhea, fever, malaria. and dengue, and in the form of compresses, applied to relieve headaches and toothaches. In addition, it is used as an antiseptic. Table 1 summarizes various parts of *T. riparia* used by indigenous populations throughout different regions of the world.

**TABLE 1:** Common traditional uses of *T. riparia* throughout different parts of the world.

Geographic location/Tribes	Pant parts	Process of preparation	Dosage and routes of administration	Disease	References
Rwanda, Tanzania, Uganda, Kenya, South Africa and Brazil	Leaves or roots or bark roots	Infusion or decoction	Oral, topical or under the tongue	Malaria, angina, cough, yaws, dropsy, helminthic diseases, stomach upsets, gastroenteritis, flatulence, mouth ulcers, toothache, gonorrhea, diarrhea, dental problems, headache and several kinds of fevers and aches, colds and flu	Hamill et al. (2000)
					Nabukenya et al. (2014)
					Nalule et al. (2011)
					Namukobe et al. (2011)
					Ngezahayo et al. (2015)
					Wasswa and Olila, (2006)
					Wintola and Afolayan, (2015)
					Van Puyvelde et al. (1986)
					Hakizamungu et al. (1988)
					Dunkel et al. (1991)
					Hakizamungu et al. (1992)
					Campbell et al. (1997)
					Gairola et al. (2009)
					Coopoosamy and Naidoo, (2011)
					Njau et al. (2014)
					de Melo et al. (2015)
					Njau and Ndakidemi, (2017)
Rwanda, West Africa	Leaves or roots	Infusion or decoction	Oral or topical	Angina, yaws, gastroenteritis, antiseptic, gonorrhea	Boily and Van Puyvelde, (1986)
Rwanda	Bark, leaves or roots	Infusion, decoction or scent of crushed leaves	Oral or inhaling the scents	Diarrhea, stomach aches, mouth ulcers, toothaches, headaches, bronchitis, influenza and swollen legs. Used as hallucinogenic herb	Chagnon, (1984)
					Van Puyvelde et al. (1985)
					Boily and Van Puyvelde, (1986)
					Van Puyvelde et al. (1988)
					Vlietinck et al. (1995)
					Gahamanyi et al. (2021)
Rwanda	Leaves	Leaves blended with banana and castor oil	—	Used as cattle medicine to repel insects and for conservation of foodstuffs in traditional silos	Van Puyvelde et al. (1987)
Rwanda	Leaves	Infusion or decoction	Oral or topical	Angina, yaws, gastroenteritis, helminths, dental abscesses, antiseptic, phagedenic ulcer, toothache, malaria, female sterility	Van Puyvelde et al. (1988)
					Gazim et al. (2010)
					Van Puyvelde et al. (1986)
					Van Puyvelde et al. (1994)
Rwanda	Leaves	Water maceration by combining leaves from three medicinal plants (*Markhamia lutea, Tetradenia riparia* and *Vernonia amygdalina*)	Oral	Malaria	Hakizamungu et al. (1992)
Western Uganda	Leaves	Squeezing by hand	Oral	Used to induce labor	Kamatenesi-Mugisha and Oryem-Origa, (2007)
Kenya	Leaf and other parts	Infusion	Oral	Treatment of boils and mumps, malaria and dengue fever	Githinji and Kokwaro, (1993)
Tanzania (the Chagga, Pare, Meru and Maasai ethnic groups from North East regions)	Leaves	Infusion and the scent	Oral	Bloody diarrhea, indigestion, constipation and malaria. The fresh leaves are used to deter houseflies and mosquitoes. Leaves are used as tonic and are boiled with beef in meat camping feasts commonly known as (olupul)	Njau, (2001)
South Africa (The Tswana people)	Leaves, shoots	Infusion	Oral	Used for fever and to calm patients, and also for gall sickness in cattle	Roberts, (1990)
South Africa and Central Africa	Leaves	Infusion	Oral	Respiratory problems, coughs, cramps, dengue, dropsy, diarrhea, angina, yaws, fever, headaches, malaria, mumps, sore throat, toothaches and treatment of gall sickness in cattle	Campbell et al. (1997)
					Hutchings, (1996)
					Duke, (2002)
					Njau and Ndakidemi, (2017)
South Africa (The Zulu people)	Leaves	Decoctions or infusions	Oral	Used to treat gastroenteritis; widely taken for cough and sore throats and as antimalarial	Van Wyk and Wink (2004)
					Njau et al. (2014)
					Bryant, (1966)
					Pooley, (1998)
South Africa	Leaves	Aqueous infusion or decoction	Taken internally and externally, as an inhalation, for headaches	Treatment against various ailments including wound healing and skin sores. Used for colds and flu, bronchitis, stomach upsets, flatulence, mouth ulcers, diarrhea, hemoptysis, fevers, malaria and headaches	Coopoosamy and Naidoo, (2011)
					Watt and Breyer-Brandwijk, 1962
Madagascar	Leaves	—	—	Cough, wounds, hepatitis	Randriamiharisoa et al. (2015)
Ethiopia	Leaves	Crushed fresh leaves homogenized in water and add salt	Oral	Diarrhea, to improve milk production of cows	Bekalo et al. (2009)

In Brazil, *T. riparia* is widely distributed in the Sao Paulo (Zelnik et al., 1978), Amazonas (Godoy et al., 1999), and Parana (Gazim et al., 2010) states, where it is popularly known as incense, lavender, lemongrass, mist plume, or false myrrh. It was introduced as an exotic ornamental plant, and is cultivated in parks, residential gardens, and vegetable gardens due to the intense and pleasant aroma it exudes. Despite this, there are no reports of its use in folk medicine in this country (Lorenzi and Matos, 2008).

## 4 Chemo-Profiling

### 4.1 *T. riparia* Leaf Extracts

**FIGURE 3:**
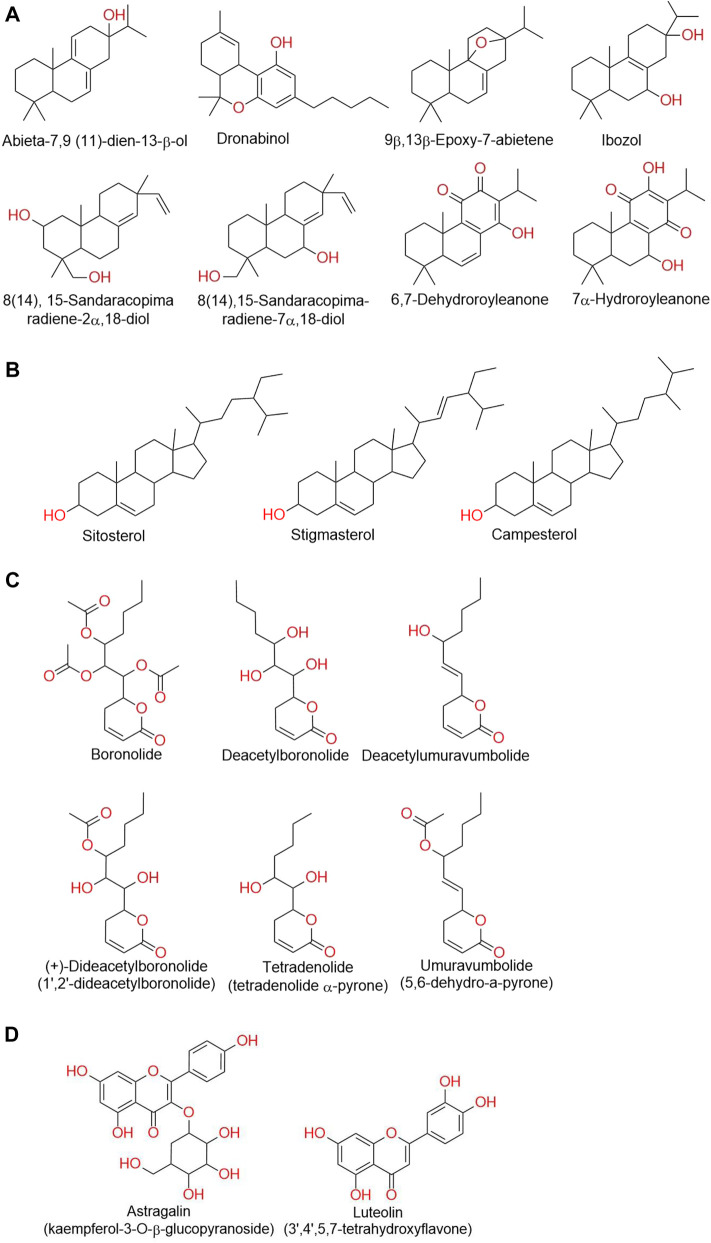
**(A)** Chemical structure of abietane and royleanone classes of isolated diterpenes from *T. riparia.* **(B)** Chemical structure of phytosterols isolated from *T. riparia*. **(C)** Chemical structure of α, β-unsaturated δ-lactone moiety-bearing phytoconstituents isolated from *T. riparia.* **(D)** Chemical structure of flavonoids isolated from *T. riparia*.

The first phytochemical investigation of *T. riparia* was carried out by Zelnik et al. (1978), from a cultivated specimens from Sao Paulo, Brazil. An extract was obtained from the dried leaves of *Iboza riparia* using acetone (Me_2_CO), and chromatographed on silica gel using hexane-benzene (C_6_H_6_) (1:1) as an eluent. The compounds 7α-hydroxyroyleanone (Figure 3A) and sitosterol were isolated initially. Further elution with a mixture of hexane-benzene:Me_2_CO (19:1) yielded the ibozol compound. By a percolation extraction technique, Van Puyvelde et al. (1981) extracted dried powder from the leaves of *T. riparia* with chloroform (CHCl_3_). The extract was filtered and evaporated *in vacuo* at 40°C (135 g), and submitted to chromatography on a silica gel column in C_6_H_6_ (C_6_H_6_-CHCl_3_-methanol (MeOH) gradient). The fractions eluted with C_6_H_6_-CHCI_3_ (25:75) contained a mixture of sterols: sitosterol (65%), stigmasterol (30%), and campesterol (5%) (Figure 3B). The fractions eluted with CHCI_3_-MeOH (93:7) yielded 1,2-dideacetylboronolide. De Kimpe et al. (1982) isolated the diterpenediol (14),15-sandaracopimaradiene-7α,18-diol from a chloroform extract obtained from the leaves of *T. riparia*. The compounds were purified by chromatography on a silica gel column in benzene (C_6_H_6_-CHCl_3_-MeOH gradient). Colorless crystals were observed after the elution of fractions with CHCl_3_-MeOH (97:3).

Van Puyvelde et al. (1987) isolated 8(14),15-sandaracopimaradiene-2α,18-diol from *T. riparia* leaves. The leaves were dried (522 g), pulverized, and extracted in a percolator with petrol (12 L). The extract was filtered, concentrated *in vacuo,* and extracted with MeOH-H_2_O (9:1). The petrol phase was then evaporated *in vacuo* to obtain a brown-green syrup, which was extracted with MeOH-H_2_O. After MeOH evaporation, the aqueous phase was extracted with CHCl_3_, resulting, after evaporation *in vacuo*, in a brown residue. Twelve g of the CHCl_3_ extract was adsorbed on silica gel, and eluted with an n-hexane-toluene-CHCl_3_-ethyl acetate (EtOAc)-MeOH gradient. The compound 8(14),15-Sandaracopimaradiene-2α,18-diol was isolated from the EtOAc fraction. Davies-Coleman and Rivett, (1995), isolated the compound 5,6-dihydro-α-pyrone (umuravumbolide) from the dry leaves from a *T. riparia* culture located in Pongola valley, Africa. The Soxhlet extract was prepared using Me_2_CO. A pale-yellow gum (0.15 g) was obtained, which showed one main spot on TLC. Flash chromatography was performed using silica gel in EtOAc-hexane (1:1), and the desacetyllumuravumbolide compound was isolated (Figure 3C).

Van Puyvelde et al. (1979) collected the leaves of *T. riparia* in the “commune” of Huye, Rwanda. The leaves were dried and pulverized and extracted with methanol (Soxhlet) for 40 h. The extract was filtered and concentrated *in vacuo* at 40°C, yielding the crude extract, which was solubilized in 2% citric acid. The suspension was filtered, defatted with petroleum ether (5 × 600 ml), and extracted with chloroform, (5 × 300 ml). The chloroform, phase was evaporated to a brown oil (6.8 g), which was chromatographed on a silica gel column in benzene (320 g with benzene-chloroform-methanol gradient). The fractions eluted with chloroform, yielded upon evaporating 1.3 g of the compound identified as umuravumbolide (5,6-dihydro-6-(3-acetoxy-1-heptenyl)-2-pyrone), a new α-pyrone from *T. riparia.* The fractions eluted with chloroform-methanol (99:l) afforded upon evaporation 1.15 g of a compound identified as deacetylumuravumbolide (5,6-dihydro-6- (3-hydroxy-1-heptenyl)-2-pyrone). The fractions eluted with chloroform-methanol (19:1) yielded upon evaporation 616 mg of a compound identified as deacetylboronolide (5,6-dihydro-6-(1,2,3- trihydroxyheptyl)-2-pyrone) (Figure 3C).

A new α-pyrone, tetradenolide, identified as 5,6-dihydro-6-(1,2-dihydroxyhexyl)-2-pyrone, was isolated from *T. riparia* leaves by Van Puyvelde and de Kimpe (1998). The authors used 713 g of dry leaves and carried out successive extractions with n-hexane and CHCl_3_. The CHCl_3_ fraction was extracted with MeOH-H_2_O (9:1). The H_2_O phase was first extracted with CHCl_3_ resulting in a CHCl_2_ extract, and then, with EtOAc. The EtOAc extract was chromatographed on silica gel and eluted stepwise in a gradient of hexane-EtOAc-MeOH. The tetradenolide compound (Figure 3C), was isolated from the fraction eluted with CHCl_3_-MeOH (19:1).

Fernandez et al. (2017) investigated the chemical composition of a crude extract obtained from the dried leaves of *T. riparia*, collected in Umuarama, Parana, Brazil. The powder obtained (230 g) was subjected to a dynamic maceration process with solvent, renewed with 70% ethyl alcohol (v/v) until the plant material was exhausted. The filtrate was then concentrated under reduced pressure in a rotary evaporator at 40 ^o^C to obtain the crude extract. Two g of crude extract was subjected to chromatography on a silica gel column, and eluted in a gradient of hexane-dichloromethane-ethyl acetate-methanol. The fraction dichloromethane-hexane (9:1) yielded the diterpene abieta-7,9 (11)-dien-13-β-ol. The fraction dichloromethane–ethyl acetate (1:1) yielded the ibozol compound. In the fraction ethyl acetate, a mixture was found of two diterpenoids: 8(14),5- sandaracopimaradiene-2α, 18-diol and 8(14),5-sandaracopimaradiene-7α, 18-diol; in the ethyl acetate–methanol (8:2) fraction, a mixture of three compounds was found. The first compound was identified as boronolide, an a-pyrone. The second compound was identified as luteolin, a flavone (Figure 3D). The third compound was identified as astragalin, a flavonol.

From the available evidence, it is clear that different groups have isolated different compounds from *T. riparia*. To what extent this is due to differences in starting material (different plant parts, differences in collection and processing), in extraction methods, or in geographical and growth conditions remains to be clarified.

### 4.2 *T. riparia* Essential Oil

#### 4.2.1 Physicochemical Characteristics

**FIGURE 4:**
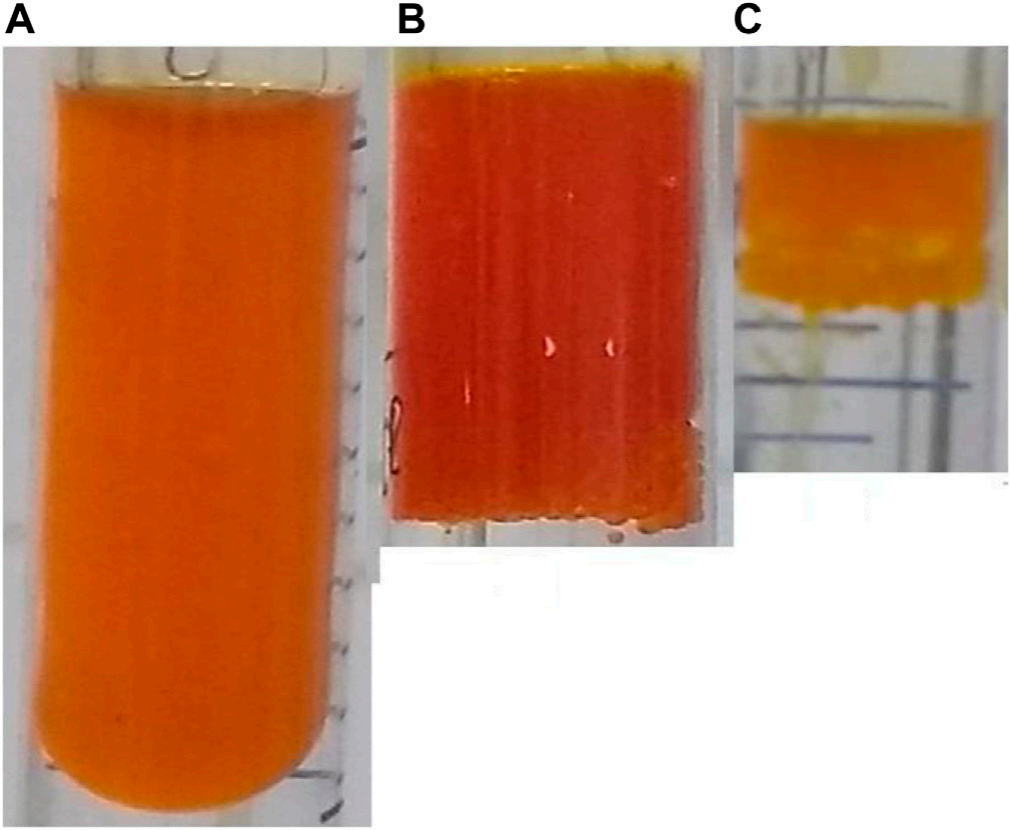
Essential oil obtained by hydrodistillation of *T. riparia* leaves **(A)**, flower buds **(B)**, and stems **(C)**—Source: Chemical Laboratory of Natural Products-Paranaense University-UNIPAR, Brazil.

The essential oil extracted from the stems of *T. riparia* has a characteristic scent of incense, and its color ranges from a light orange (Figure 4), intensifying to a darker orange in the leaves (Figure 4), to a reddish-orange in the flower buds (Figure 4).

Physicochemical analysis was standardized, and the quality of essential oils was evaluated. The determination of density, refractive index, and rotating power are parameters used to detect adulterations in the essential oils (Gil, 2007). The determination of essential oils yield within the plant is essential for carrying out biological tests, its application in products, and commercialization. According to the European Pharmacopoeia, the minimum extraction yield of essential oils for the development of products and application is 2 ml/kg (European Pharmacopoeia, 2013). Physicochemical indexes of *T. riparia* essential oil are shown in Table 2.

**TABLE 2:** Physicochemical indexes of *T. riparia* essential oil.

Localization	Parts	Physico-chemical indexes					References
—	—	Refraction index $n_{D}^{20}$		Specific rotation ${\left|\alpha \right|}_{D}^{20}$	Relative density (g/ml) $d_{20}^{20}$	Yield (%)	
Butari, Rwanda	Leaves	—		—	0.92	—	Weaver et al. (1994)
Kirstenbosch National Botanic Gardens, Cape Town	Leaves	14,685		+6.4°	08,874	—	Campbell et al. (1997)
Manaus, Amazonas, Brazil	Leaves	—		—	—	0.39	Godoy et al. (1999)
Botanical Garden Umuarama, Parana, Brazil	Leaves	—	—		—	0.265 ± 0.0	Gazim et al. (2010)
Medicinal Garden –EMATER Goiania, Goias, Brazil	Leaves	—	—		—	0.17 ± 0.05	Araújo et al. (2018)
Greenhouse South Mississippi Branch Poplarville, MI, United States	Aerial parts	—	—		—	0.80	Blythe et al. (2020)
Medical Garden Umuarama, Parana, Brazil	Leaves	—	—		—	0.29 ± 0.22	Zardeto-Sabec et al. (2020)
	Flower buds	—	—		—	0.38 ± 0.17	—

The same study by Gazim et al. (2010) analyzed the yield (g %) of the essential oil under different climatic seasons; the lowest oil yield occurred in plants harvested in spring (0.168 g ± 0.02), and the highest yield was observed in plants harvested in winter (0.265 g ± 0.025), whereas the oil content of plants harvested in summer and autumn remained close, (0.215 g ± 0.007 and 0.237 g ± 0.011, respectively).

#### 4.2.2 Chemical Composition of Essential Oil

**FIGURE 5:**
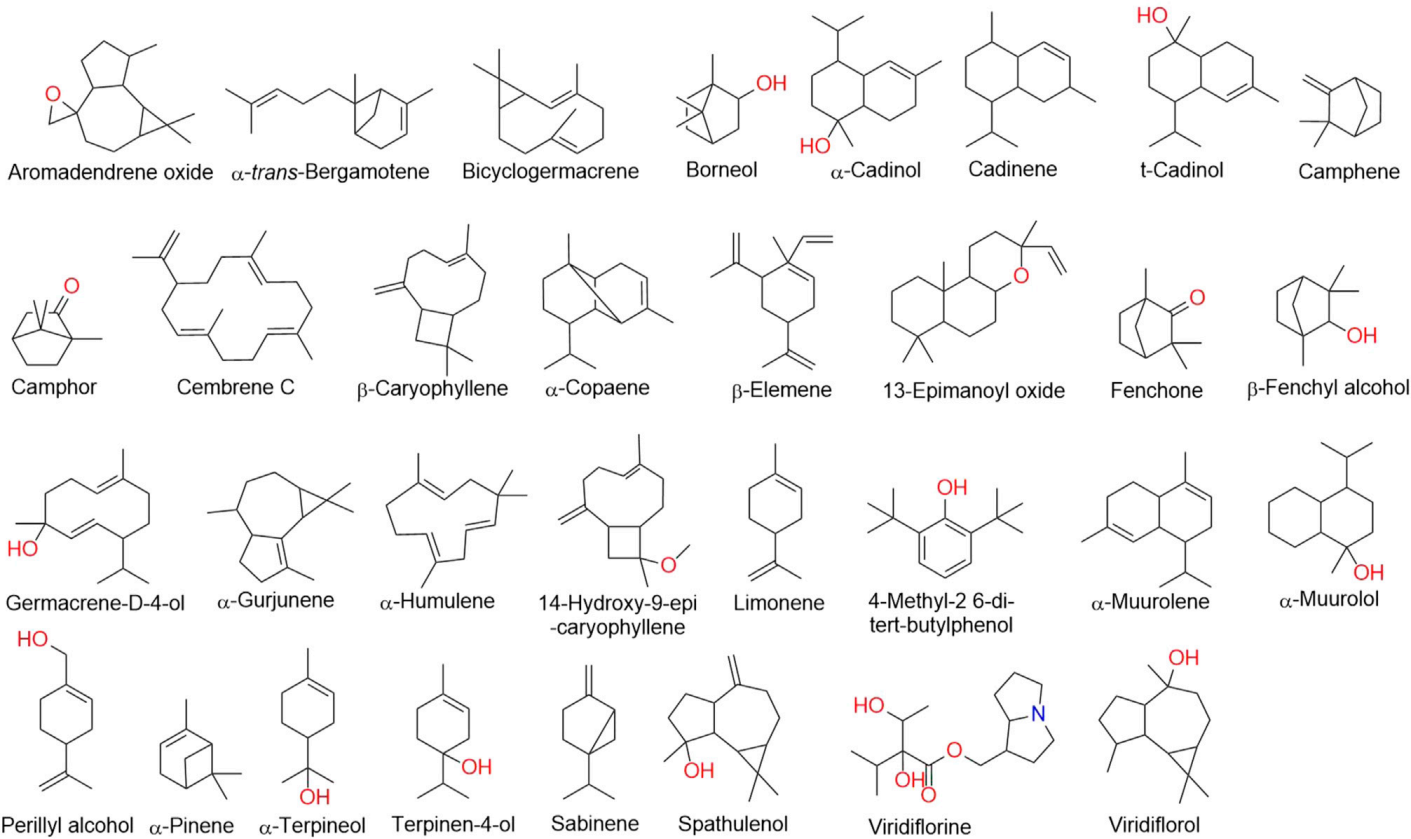
Chemical structures of volatile constituents isolated from *T. riparia*.

*T. riparia* essential oil has a complex composition of terpenoids, with numerous compounds present in small concentrations. Terpenes are the majority class, represented by monoterpene, sesquiterpene, and diterpene hydrocarbons and oxygenates (Weaver et al., 1994). However, the concentration of these terpenoids varies according to factors such as the location of the crop implantation, climate, altitude, soil, and collection time. Therefore, we reviewed the literature to compare the chemical composition of *T. riparia* essential oil from different locations: Africa (South Africa and Kenya) (Campbell et al., 1997; Omolo et al., 2004), South America (Brazil) (Godoy et al., 1999; Gazim et al., 2011 and, 2014; Araújo et al., 2018), and North America (Poplarville, MI, United States) (Blythe et al., 2020); and the chemical identification of the essential oil is shown in Table 3 and Figure 5.

**TABLE 3:** Chemical Composition of *T. riparia* essential oil.

Localization	Parts	Extraction and analysis technique	Essential Oil chemical composition	References
Kirstenbosch National Botanic Gardens, Cape Town	Aerial parts (leaves and stems) of *T. riparia*	Hydrodistillation (1 h) and evaluated by GC/MS.	Monoterpenes were the predominant class of compounds (69.0%). The major compounds were α-terpineol (22.6%); fenchone (13.6%); fenchyl alcohol (10.7%), and β-caryophyllene (7.9%)	Campbell et al. (1997)
Nyanza, Western, Rift Valley, and Central provinces of Kenya	Leaves	Hydrodistillation and evaluated by GC/MS.	Oxygenated monoterpenes were the predominant class (66.45%); The major compound was fenchone (64.82%)	Omolo et al. (2004)
Manaus, Amazonas, Brazil	Leaves	Hydrodistillation for 5 h and evaluated by GC/MS.	Oxygenated monoterpenes were the predominant class (28,3%), followed by oxygenated sesquiterpenes (22.0%). The major compounds were fenchone (19.9%); 14-hydroxy-9-epi(E)-caryophyllene (12.3%); α-cadinol (5.2%); isocaryophyllene (3.9%); camphor (3.4%) and σ-cadinene (3.1%)	Godoy et al. (1999)
Botanical garden of Paranaense University, Umuarama, Parana, Brazil	Leaves	Hydrodistillation for 3 h and evaluated by GC/MS.	Oxygenated sesquiterpenes were the predominant class (64.30%). The major compounds were 14-hydroxy-9-epi-caryophyllene (18.03%); *cis*-muurolol-5-en-4-a-ol (11.73%); ledol (7.18%); α-cadinol (4.90%); The second class was that of oxygenated monoterpenes (20.6%). The major compounds were limonene (3.69%) and fenchone (12.87%)	Gazim et al. (2011)
Botanical garden of Paranaense University, Umuarama, Parana, Brazil	Leaves	Hydrodistillation for 3 h. Chromatography on a silica gel support and eluted with a pentane–dichloromethane–methane gradient. Analysis by NMR	In the pentane-dichloromethane (9:1) fraction, white crystals were isolated and identified as 9β,13β-epoxy-7-abietene; In the pentane-dichloromethane (8:2) fraction, orange crystals were isolated and identified as 6,7-dehydroroyleanone	Gazim et al. (2014)
Biological Science Institute of Goias Federal University, Goiania, Brazil	Leaves	Hydrodistillation GC/MS.	Oxygenated sesquiterpenes were the predominant class (21.52%). The major compounds were 14-Hydroxy-9-epi-(E)-caryophyllene (16.03%). The second class was that of oxygenated monoterpenes (11.32%), and the major compound was fenchone (7.90%)	Araújo et al. (2018)
Greenhouse located in the South Mississippi Branch Experiment Station in Poplarville, MI, United States	Leaves and stem	Hydrodistillation (3 h)	The predominant class was that of oxygenated sesquiterpenes (29.30%), with 14-Hydroxy-β-caryophyllene (7.9%) and tau-cadinol (6.9%) as the major components. The hydrocarbons sesquiterpenes were the second most abundant class (28.4%), and the major compounds were δ-cadinene (10.6%); followed by the oxygenated monoterpene fenchone (14.8%)	Blythe et al. (2020)
Botanical garden of Paranaense University, Umuarama, Parana, Brazil	Leaves, flower buds and stems	Hydrodistillation for 3 h	In flower buds there was a predominance of oxygenated sesquiterpenes (43.62%) with α-cadinol (13.69%) and 14-hydroxy-9-epi-caryophyllene (15.38%) as the major components. In the leaves, hydrocarbon sesquiterpenes dominated (26.44%), with α-cadinol (12.21%) as the main component. Oxygenated monoterpenes were the second most abundant class (16.44%) with fenchone (11.57%) as the major compound	Zardeto-Sabec et al. (2020)

##### 4.2.2.1 Chemical Composition and Seasonal Variation

Two studies were conducted to evaluate the influence of abiotic factors on the chemical composition of *T. riparia* essential oil. The first, carried out by Araújo et al. (2018), evaluated the influence of the level of shading on the chemical composition of *T. riparia* leaves essential oil. The authors demonstrated that the concentrations of the sesquiterpene hydrocarbon 14-hydroxy-9-epi (E)—caryophyllene and the oxygenated monoterpene fenchone were influenced by the level of shading where the plant is growing. Two compounds were identified in all different levels of light applied; 14-hydroxy-9-epi (E)—caryophyllene and fenchone. The former was the major compound (16.48%) in plants grown under 30% shading, followed by those grown under 80% shading (16.42%); plants grown under 50% shading or full sunlight showed the lowest content of this component with 16.41% and 16.03%, respectively. Fenchone content was the highest (9.93%) in plants grown under 30% shading, followed by those grown in full Sun, 50% or 80% shading, with levels of 7.90, 7.78, and 3.59%, respectively.

Gazim et al. (2010) evaluated the chemical composition of *T. riparia* essential oil collected in spring, summer, autumn, and winter in the northwest region of the Parana State, Brazil. Leaves collected in winter have a higher percentage of calyculone (24.70%), abietadiene (13.54%), and viridiflorol (4.20%). Leaves collected in autumn showed higher percentages of ledol (8.74%) and cis-muurolol-5-en-4-α-ol (13.78%), and leaves collected in spring or summer had higher percentages of fenchone (12 0.67%), 14-hydroxy-9-epi-caryophyllene (24.36%), and α-cadinol (8.33%).

## 5 Pharmacology and Bioactivity

### 5.1 Antimicrobial Activity of Crude Extract

York, et al. (2012), investigated the antimicrobial effects of 30 plant species, among them *T. riparia,* used for the treatment of respiratory infections by the population of a rural region of Maputaland, Kwazulu-Natal, South Africa. *In vitro* minimal inhibitory concentration (MIC) assays were performed with the organic extract (dichloromethane: methanol), aqueous extract and essential oil obtained from the leaves of *T. riparia*. These were tested against *Cryptococcus neoformans* (ATCC 14116), *Klebsiella pneumoniae* (ATCC 13883), *Moraxella catarrhalis* (ATCC 23246), *Mycobacterium smegmatis* (ATCC 14468) and *Staphylococcus aureus* (ATCC 6538). The organic extract and essential oil were active against fungi *Cryptococcus neoformans* with MIC of 0.60 mg/ml and 0.83 mg/ml, respectively. The organic extract was also active against *Moraxella catarrhalis* (MIC of 0.10 mg/ml) and *Staphylococcus aureus* (MIC of 0.03 mg/ml).

Fernandez et al. (2017) investigated the antibacterial potential of crude extract and fractions of *T. riparia* leaves by a broth microdilution method. The crude extract was fractionated, and the ibozol compound isolated from the dichloromethane:ethyl acetate (1:1) fraction. This compound showed high activity against *S. aureus* (MIC of 1.95 μg/ml). From the ethyl acetate fraction, 8(14),5-sandaracopimaradiene-2α, 18-diol and 8(14),5-sandaracopimaradiene-7α,18-diol were isolated, which showed activity against the *S. aureus* (0.98 μg/ml) and *Enterococcus faecalis* and *Bacillus cereus* (31.2 μg/ml).

Kakande et al. (2019), evaluated the antifungal potential of a *T. riparia* leaves crude alcoholic extract against the fungi *Trichophyton tonsurans*, *Trichophyton mentagrophyte*, and *Microsporum audouinii*; they found MICs ranging from 62.5 to 250 mg/ml, and a minimum fungicidal concentration (MFC) ranging from 125 to 500 mg/ml. This expands the range of pharmacological applications of *T. riparia*, indicating potential for the control of *Trichophyton,* which causes skin, nails, and hair dermatophytosis.

Elaka et al. (2020), investigated the antibacterial potential of a *T. riparia* leaves dichloromethane: methanol (1:1) extract. The extract was obtained by percolation, and tested against *S. aureus* ATCC 25923, *E. coli* ATCC25922, and *P. aeruginosa* ATCC 9027). The strongest activity was found against *E. coli* (MIC of 125 μg/ml).

Friedrich et al. (2020) studied strawberries coated with a film based on cassava starch, gelatin, and sorbitol, and containing *T. riparia* leaves crude extract at concentrations ranging from 500 to 1,000 μg/ml. Incorporation of the crude extract into the film inhibited the development of microbial colonies by 98% over 5 days, thus indicating the ability of the crude extract to preserve stored strawberries.

### 5.2 Antimicrobial Activity of Essential Oil

The essential oil from *T. riparia* leaves harvested in the summer showed activity against *S. aureus* (MIC of 15.6 μg/ml), *B. subtilis* (7.8 μg/ml), *E. faecalis* (62.5 μg/ml), *P. aeruginosa* and *E. coli* (both 125 μg/ml) as well as antifungal activity against *Candida albicans* (31.2 μg/ml) (Gazim et al., 2010).

Baldin et al. (2018) analyzed the anti-*Mycobacterium tuberculosis* activity of the essential oil and the isolated compound 6,7-dehydroroyleanone obtained from *T. riparia* leaves. Both showed activity against *Mycobacterium tuberculosis* H37Rv, and a similar MIC was found for clinical isolates (between 31.2 and 62.5 μg/ml).

In a study carried out by Scanavacca et al. (In Press), *T. riparia* leaves essential oil showed high antimicrobial activity against all bacteria evaluated, with MICs ranging from 0.05 to 0.60 mg/ml, similar to the MIC for streptomycin (0.05–0.125 mg/ml) and ampicillin (0.10–0.30 mg/ml). Gram-positive bacteria showed greater sensitivity to the essential oil: *B. cereus* (0.05 mg/ml), *Listeria monocytogenes* (0.05 mg/ml), and *S. aureus* (0.05 mg/ml), while *P. aeruginosa* was less sensitive (MIC of 0.60 mg/ml). The essential oil also inhibited the growth of fungal strains with MICs ranging from 0.06 to 10.0 mg/ml, which is more potent than the positive controls bifonazole (0.10–0.20 mg/ml) and ketoconazole (0.15–2.50 mg/ml). The most sensitive fungus was *Aspergillus versicolor* (0.06 mg/ml), followed by *Penicillium ochrochloron* (0.50 mg/ml).

### 5.3 Antimicrobial Activity With Special Reference to Multi Drug-Resistant Pathogens

#### 5.3.1 Antimycobacterial Activity

Van Puyvelde et al. (1994) studied the antimycobacterial activity of 8(14),15-sandaracopimaradiene, 7α,18-diol isolated using bioassay-guided purification from leaves of *T. riparia* (MIC, 25–100 μg/ml). Later, Baldin et al. (2018) also obtained a similar MIC value (31.25 μg/ml) against *Mycobacterium tuberculosis* H37Rv and several resistant clinical isolates for a different compound (the diterpene 6,7-dehydroroyl) from the essential oil. The compound 6,7-dehydroroyleanone displays moderate activity against multidrug-resistant isolates, with little cytotoxicity to murine macrophages.

#### 5.3.2 Anti-Dermatophytic Activity

**FIGURE 6:**
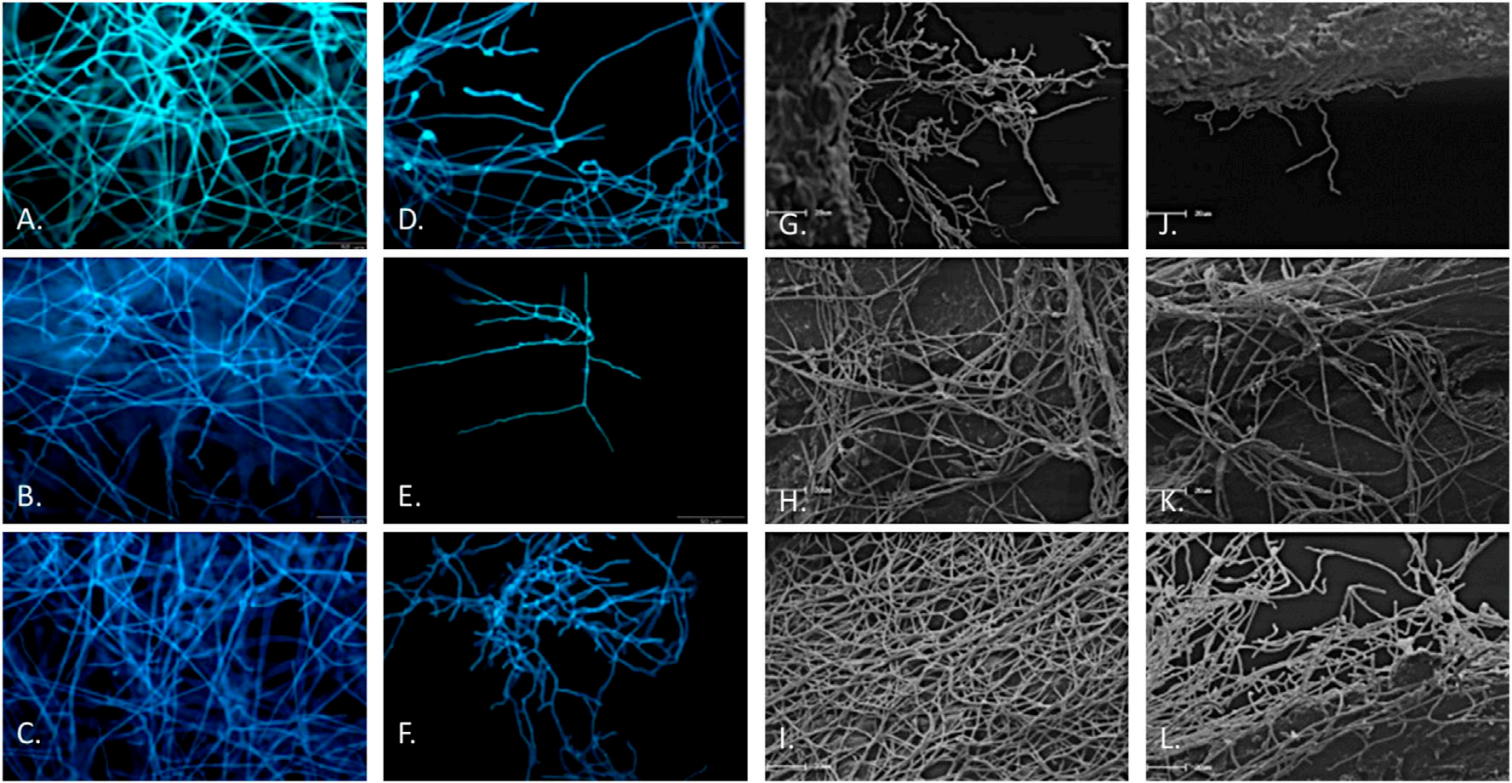
Fluorescence microscopy. **(A–C)** Control cells of *T. rubrum, T. mentagrophytes* and *M. gypseum*, respectively. **(D–F)** *T. rubrum*, *T. mentagrophytes* and *M. gypseum* treated with 31.2, 62.5, and 31.2 μg/ml of *T. riparia* extract, respectively. Scanning Electron Microscopy, **(G–I)** Control cells of *T. rubrum, T. mentagrophytes* and *M. gypseum*, respectively. **(J–L)** *T. rubrum*, *T. mentagrophytes* and *M. gypseum* treated with one-fold sub-MIC concentrations of T. riparia extract (adapted from Endo et al., 2015 ^1^).

Endo et al. (2015) tested T. riparia crude extract against several dermatophytes (*Trichophyton rubrum*, T. mentagrophytes and *Microsporum gypseum*) using a microdilution method (MIC and MFC) as well as (fluorescence and scanning electron) microscopy. Hydroalcoholic leaf extract showed strong activity against all three test strains (MIC ranging from 62.5 to 125 μg/ml while MFC 62.5–250 μg/ml). Concentrations of 31.2 and 62.5 μg/ml caused a reduction in hyphal growth, and irregular patterns as well as ungerminated conidia were observed with fluorescence microscopy (Figure 6). Strong inhibition of hyphal growth with irregular growth patterns were also observed using scanning electron microscopy (Endo et al., 2015).

##### 5.3.2.1 Anti-Biofilm Activity

*T. riparia* extract had strong effects against pre-formed *S. aureus* (both methicillin resistant-MRSA and methicillin sensitive-MSSA) biofilms, with BIC_50_ values of 30–90 μg/ml, which were less than the MIC values of 31.2–125 μg/ml. SEM micrographs showed a strong reduction of the number of cells and disruption of organized structure of *S. aureus* ATCC 29213 biofilms when treated at a concentration of 250 μg/ml (Endo et al., 2018).

Costa et al. (2015) found that an hydroalcoholic extract of *T. riparia* inhibited *C. albicans* biofilm at a concentration of 62.5 μg/ml which was more effective compared with standard fluconazole (MIC >1,000 μg/ml). while less effective than nystatin (MIC = 7.8 μg/ml).

Van Puyvelde et al. (2021) reported the activity of dichloromethane and ethyl acetate fractions obtained from *T. riparia* leaves extracts against foodborne pathogens *Shigella sonnei, Salmonella enterica, E. coli, Micrococcus luteus, S. aureus, and E. faecalis*. In the dichloromethane fraction, the compound 8(14),15-sandaracopimaradiene-7α,18-diol was identified, which showed activity against bacteria with IC_50_ ranging from 11.2 to 212.5 μg/ml. In the ethyl acetate fraction, the compounds deacetylumuravumbolide and umuravumbolide were found, which showed a moderate activity with IC_50_ between 212.9 and 637.7 μg/ml and 176.1–521.4 μg/ml, respectively. This study reported that 8(14),15-sandaracopimaradiene-7α, 18-diol is bactericidal against *S. aureus*, and also has antibiofilm activity (BIC_50_, 8.8 ± 1.5 μg/ml) similar to planktonic activity (MIC_50_, 11.4 ± 2.8 μg/ml).

##### 5.3.2.2 Synergy Studies

Endo et al. (2018) combined a hydroalcoholic extract from the leaves of *T. riparia* with the standard drug penicillin against 13 multidrug resistant/sensitive *S. aureus* strains and found synergistic effects against 69.2% of the isolates. From the checkerboard assay, synergy was found with five isolates such as MRSA 78 (Fractional Inhibitory Concentration Index, FICI = 0.14), MRSA 81 (FICI = 0.24), MRSA 83 (FICI = 0.18), MSSA 97 (FICI = 0.26), and MSSA 170 (FICI = 0.25). Although this is a very interesting findings, the authors did not identify which bioactive plant compounds are responsible for the synergy. Later, Van Puyvelde et al. (2021) useded bioassay-guided purification with *S. aureus* as model organism, and obtained 8(14),15-sandaracopimaradiene-7α, 18-diol as the major compound responsible for the bactericidal as well as antibiofilm activity. This research needs to be followed up further to combine this compound with penicillin to study synergy. Costa et al. (2015) also observed synergy when *T. riparia* extract was combined with nystatin against *C. albicans* (FICI = 0.24). This was also a preliminary study, which needs further exploration with the active anticandidal plant compounds combined with various antifungal agents. Supplementary Table S1, summarizes list of antimicrobial compounds reported from various studies, including isolation and identification techniques.

### 5.4 Anti-tumor and Antioxidant Activity of Essential Oil From *T. riparia* Leaves

Essential oil from *T. riparia* leaves and two compounds (9β, 13β-epoxy-7-abieethane and 6,7-dehydroroileanone) were evaluated for cytotoxic potential by a 3-(4,5-dimethylthiazol-2-yl)-2,5-diphenyl-2H-tetrazolium bromide (MTT) assay, using tumor cells MDA-MB-435 (human breast carcinoma), HCT-8 (human colon), SF-295 (human glioblastoma) and HL-60 (human promyelocytic leukemia). The essential oil and compound 9β, 13β-epoxy-7-abietane showed high cytotoxic activity on the cell lines SF-295 (78.06 and 94.80%), HCT-8 (85.00 and 86.54%), and MDA -MB-435 (59.48 and 45.43%). Moreover, the cytotoxicity of the essential oil and the isolated compound yielding a selectivity index (SI) of 1.9 for the essential oil and 7.9 for 6,7-dehydroroyleanone. The selectivity index was defined as the ratio between the cytotoxicity of the compound for tumor cells and its activity on non-tumor mammalian cells (VERO). The higher this index, the greater the specificity of these molecules for the tumor cells tested, thus indicating grater specificity of the isolated compound 6,7-dehydroroyleanone for tumor cells.

The authors also investigated the antioxidant activity of the essential oil and isolated compounds by the 2,2-diphenyl-1-picryl-hydrazyl (DPPH) and β-carotene-linoleic acid assays. The compound 6,7-dehydroroileanone strongly regenerated the DPPH radical with IC50 = 0.01 μg/ml. The essential oil and 6,7-dehydroroileanone also inhibited oxidation in the β-carotene linoleic acid test with 130.1% for the essential oil and 109.6% for the compound 6,7-dehydroroileanone. Thus, 9β, 13β-epoxy-7-abietene showed high cytotoxic potential and 6,7-dehydroroileanone high antioxidant potential (Gazim et al., 2014).

### 5.5 Analgesic Activity of *T. riparia* Leaves Essential Oil

Analgesic effects were studied of the essential oil from *T. riparia* leaves harvested during different seasons: spring, summer, autumn, and winter. An oral dose of 200 mg/kg caused analgesia in mice, inhibiting acetic acid-induced contortions by 38.94–46.13%. This effect showed no seasonal variation (Gazim et al., 2010).

### 5.6 Antiparasitic Activity of *T. riparia* Leaves

The vermicidal action of essential oil from *T. riparia* leaves was investigated by de Melo et al. (2015) at two concentrations: 50 and 100 μg/ml on *Schistosoma mansoni* worms. All worms died after 24 h of incubation with 100 μg/ml. At 50 μg/ml, the oil reduced the motor activity of the adult worm after periods of more than 72 h of incubation. Also, after 120 h of incubation, there was a slight decrease in the number of eggs produced by adult *Schistosoma mansoni* worms and a dose-dependent reduction in egg development.

The leishmanicidal action of the essential oil from the leaves of *T. riparia* was investigated by Cardoso et al. (2015). The oil inhibited the growth of *Leishmania* (*L*.) *amazonensis* promastigotes after 24 h of treatment. The inhibitory concentrations (IC_50_) were 15.47 ± 4.6 ng/ml for oil samples obtained in the spring, 15.67 ± 1.70 ng/ml in the summer, 15.66 ± 2.22 in the autumn and 13.31 ± 0.85 ng/ml in the winter.

In the same study, the essential oil of *T. riparia* obtained in different seasons also inhibited the survival of intracellular *L. amazonensis* amastigotes at concentrations of 30 (*p* < 0.001) and 3 ng/ml (*p* < 0 0.05). The strongest effects were observed at concentration of 30 ng/ml, with inhibition of parasite growth of 43.53, 32.03, 40.54, and 52.49% for the oils obtained in spring, summer, autumn, and winter, respectively (Cardoso et al., 2015).

**FIGURE 7:**
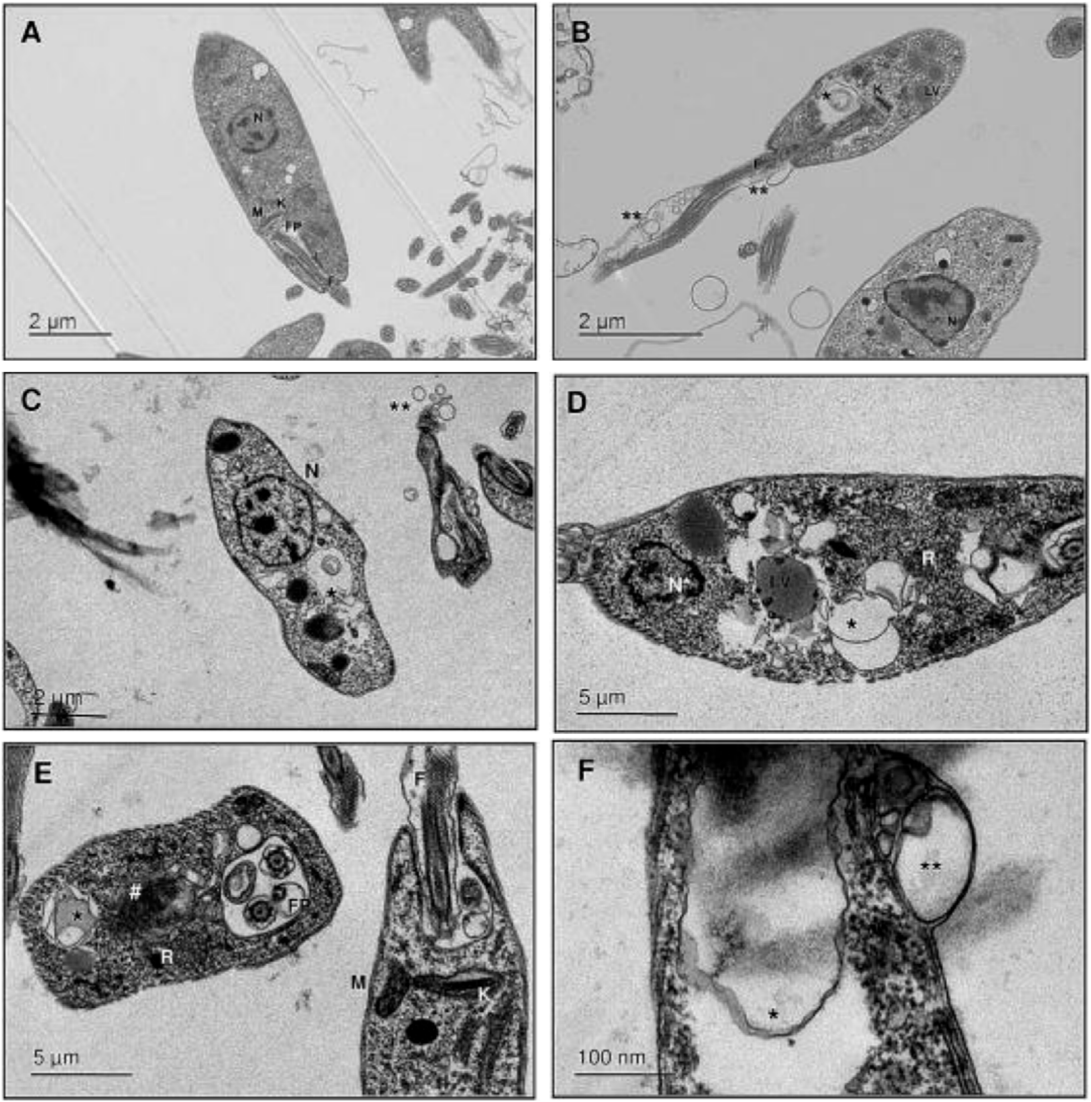
Transmission electron microscopy of L. amazonensis treated with *T. riparia* essential oil (TrEO) for 24 h. **(A)** *Leishmania* promastigotes. **(B–F)** Promastigotes treated with TrEO (30 ng/ml). N, nucleus; N*, abnormal chromatin condensation nuclear alterations; K, kinetoplast; M, mitochondria; FP, flagellar pocket; F, flagellum; V, vacuoles; LV, lipid vesicles; R, myelin-like figure appears in close association with the flagellar pocket membrane; *membranous profiles; **blebbing; ^#^mitochondrial swelling (Adopted from Demarchi et al., 2015 ^2^).

Another study with *L. amazonensis* demonstrated that 30 ng/ml of the essential oil from *T. riparia* leaves induced 50% amastigote death after 24 h of incubation. For the infected and untreated macrophages, the infection index was 112 for each macrophage (Demarchi et al., 2015), with an effective concentration of 30 ng/ml (IC_50_) and an LD_50_ of 0.5 μg/ml. Transmission electronic microscopy revealed modifications of the morphology of *L. amazonensis* promastigotes with ultrastructural changes such as “cytoplasm vacuolization, membranous profiles inside the organelle, lipid vesicles, and membrane blebbing that suggested autophagy, thickening of the kinetoplast, chromatin condensation, and nuclear fragmentation” (Figure 7) (Demarchi et al., 2015). The same study, demonstrated that the essential oil of *T. riparia* had no cytotoxicity in murine macrophages at 30 ng/ml (>95% viable cells); however, 0.2 μg/ml had a cytotoxic effect of 50%. The authors suggest that the essential oil in high doses is cytotoxic to macrophages, while lower doses are effective against the parasite. Thus, the dose of the essential oil and the route of administration need to be evaluated under specific conditions.

*T. riparia* essential oil stimulated pro-inflammatory cytokine expression in macrophages; the effects varied according to the incubation time. After 3 h, the mRNA expression of interleukin-1β (IL-1β), IL-10, IL-12, IL-17, and IFN-γ was detected, but only IL-1β expression remained high after 6 h. Tumor necrosis factor α (TNF-α), IL-18, and IL-33 mRNA expression was unchanged at 3 or 6 h. The essential oil modulated the synthesis of cytokines up to 24 h. Cytokine production, e.g. of IL-10, IL-4 and IL-5, was also induced by infection with *L. amazonensis*, which may be prevented by treatment with the essential oil. Cell proliferative mediators and granulocyte-macrophage colony-stimulating factors were also inhibited by treatment with *T. riparia* essential oil. The results suggest that the essential oil reduced the expression of cytokines related to the infection progress, and increased IFN-γ (Demarchi et al., 2015).

Lorenzi and Matos (2008) showed that the essential oil from *T. riparia* leaves has moderate antimalarial activity against the malaria parasite *Plasmodium falciparum*.

Van Puyvelde et al. (2018), isolated 8(14), 15-sandaracopimaradieno-7α, 18-diol from *T. riparia* leaves, and found anthelmintic activity against the model nematode *Caenorhabditis elegans* (wild type and mutants) with an IC_50_ of 5.4 ± 0.9 μg/ml. The anthelmintic activity of this diterpenediol validates the use of *T. riparia* for worm infection by Rwandese tribes in Africa. Moreover, 8(14),15-sandaracopimaradiene-7α,18-diol had similar potency against a Slo-1 mutant of *C. elegans* (Slo-1 is an orthologue of mammalian BK channels), suggesting that this channel is not the molecular target.

### 5.7 Insecticide and Acaricide Activity of the Essential Oil From *T. riparia* Leaves

Lorenzi and Matos (2008) showed that the essential oil from *T. riparia* leaves has insect repellent action, in particular against the species *Anopheles gambiae*.

Fernandez et al. (2014) analyzed the activity of *T. riparia* leaves essential oil against *Aedes aegypti* larvae, using essential oil obtained at different times (spring, summer, autumn and winter). Larvae were exposed for 24 h to concentrations ranging from 2,500 to 3,125 μg/ml, and the larvicidal activity was measured by calculating the lethal dose (LD_50_) using the Probit test. Larvicidal activity (mainly the LD_50_) varied with the seasons (78.72; 83.29 and 123.02 μg/ml for autumn, spring and summer, respectively; and lowest larvicidal activity in winter (2,619.79 μg/ml).

The percentage mortality of *Rhipicephalus* (*Boophilus*) *microplus* mite larvae exposed to different concentrations of essential oil from *T. riparia* leaves was measured by the larval immersion test (LIT). At concentrations of 100, 50, and 25% the essential oil showed maximum efficacy with a mortality rate of 100% of the larvae. At dilutions ranging from 12.5% to 0.014%, larval mortality ranged from 97.6 to 10.60%, respectively (Gazim et al., 2011).

Zardeto-Sabec et al. (2020) evaluated the essential oil of *T. riparia* leaves or flower buds against *Rhipicephalus sanguineus* tick larvae. Larvae were exposed for 24 h to concentrations ranging from 50,000–0.47 mg/ml, and the larvicidal activity was measured by calculating lethal concentration (LC) using the Probit test. The LCs of the oils that killed 99.9% of the larvae (LC_99.9_) were 9.98 ± 0.10 mg/ml for the essential oil of the leaves, and 20.12 ± 0.54 mg/ml for that of the flower buds. The authors also studied the mechanism of action of the essential oil, evaluating the inhibitory potential on the enzyme acetylcholinesterase (AChE), whose inhibition doses were 0.70 mg/ml for the essential oil of the leaves and 1.40 mg/ml for that of the flower buds. The insecticidal action of *T. riparia* leaves essential oil was evaluated by Weaver et al. (1994). The oil was diluted at concentrations of 396, 791, 1,583, and 3,165 μg/cm^2^, applied to filter paper, and placed inside a Petri dish containing adult *Zabrotes subfasciatus* insects (0–2 days post-emergence from the bean, 5 males and 5 females). Insects that were moribund or died after oil exposure within 24 h were pooled to calculate the percentage of incapacitation by Probit analysis. The authors also sprayed the oil at different concentrations on beans containing eggs and larvae of *Z. subfasciatus*. The essential oil interfered with the reproduction of adult females (EC_50_ of 72 μg/cm^2^). Eggs were also sensitive to oil with an EC_50_ of 50 μg/cm^2^. The activity of the oil on the larvae was lower, as they were inside the bean grains, making it difficult for the oil to penetrate, with an EC_50_ of approximately 3,980 μg/cm^2^. These results validate the popular use of *T. riparia* leaves in grain storage silos, helping the preservation of grains during storage.

This bibliographic review verified that *T. riparia* has wide biological activities. Although investigations began 50 years ago, there is still much to learn about this plant. Its flower buds and stems have not yet been thoroughly investigated from a phytochemical and bioactivity point of view, opening new perspectives for biological assays.

## 6 Computational Investigation (*in Silico* Studies)

Although several compounds have been isolated from *T. riparia,* the bioactivity of only a few were documented. Only five compounds were identified through bioassay-guided purification based on multiple biological activities. In an attempt to close this gap, molecular docking was employed to assess potential antibacterial (anti-biofilm), anticancer (anti-inflammatory) and antiparasitic (anti-Leishmania) mechanisms using three putative target enzymes: the biofilm-associated *Staphylococcus aureus* sortase A (SaSrtA) (Thappeta et al., 2020), the inflammatory and cancer-associated human cyclooxygenase-2 (hCOX-2) (Méric et al., 2006) and the *Leishmania infantum* trypanothione reductase (LiTH) as an antiparasitic target (Saccoliti et al., 2017). The crystallographic structure of each enzyme was retrieved from the protein data bank (PDB): SaSrtA (PBD ID: 1T2W), hCOX-2(PDB ID: 5IKT) and LiTH (PDB ID: 2JK6). All twenty isolated phytochemicals from *Tetradenia* were used as ligands for docking study. Three-dimensional structures of both target and ligand were saved in .pdb file format for virtual screening with the software PyRx-AutoDock (Swain et al., 2021a). The protein-ligand interactions were visualized using Discovery Studio Visualizer software (Sahoo et al., 2021). Furthermore, the structural-activity relationship (SAR) between each phytochemical and its biological activity was explored mainly the software ChemDraw 18.0 (Swain et al., 2021b).

**FIGURE 8:**
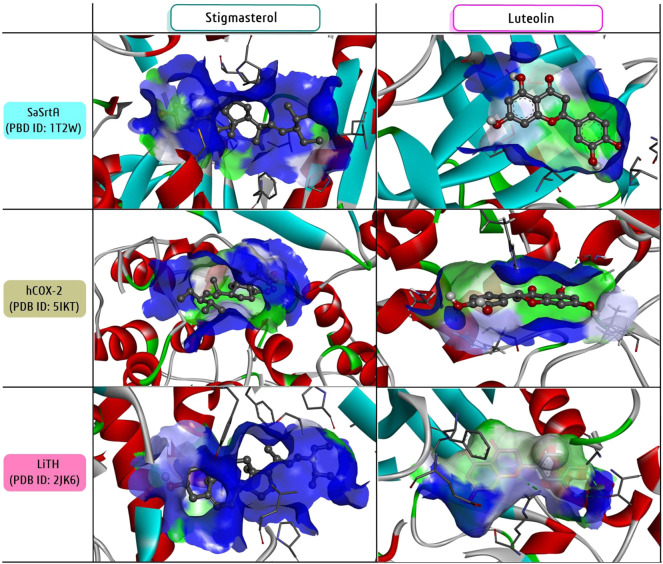
Protein-ligand interactions of two most potential candidates, stigmasterol and luteolin against selected three candidates. The molecular interactions were presented using the software Discovery studio visualizer.

The docking score (kcal/mol) of each phytochemical against the three individual target enzymes was recorded (Table 4 and Figure 8). All phytochemicals exhibited docking scores between −5 and −9 kcal/mol. All three-sterol classes of compounds, campesterol (−7.8 kcal/mol), stigmasterol (−7.8 kcal/mol), sitosterol (−7.6 kcal/mol) and flavonoid luteolin (−7.5 kcal/mol) had higher scores than other terpenes against SaSrtA. On the other hand, astragalin and luteolin (−8.4 kcal/mol) together with stigmasterol and 13-epimanoyloxide (−8 kcal/mol) exhibited the highest scores against hCOX-2. Finally, stigmasterol (−9 kcal/mol), astragalin (−7.9 kcal/mol), luteolin (−7.6 kcal/mol) and 9β,13β-epoxy-7-abietene (−7.3 kcal/mol) showed the highest docking scores against LiTH. Based on the average docking score against three targets, stigmasterol (−8.26 kcal/mol), luteolin (−7.83 kcal/mol), astragalin (−7.66 kcal/mol), sitosterol (−7.53 kcal/mol) were the four most potent candidates, and their interactions with the three potential target enzymes are shown in Figure 8. However, often the docking score of a compound shows little difference between the three (unrelated) targets (Table 4), suggesting that the predicted interaction is rather non-selective. Nonetheless, bioinformatics tools play an increasing role as a guide in contemporary drug discovery and development by assessing possible biological activity targeting. This helps to reduce time and resources devoted to experimental testing (Romano and Tatonetti, 2019; Sahoo et al., 2021).

**TABLE 4:** Molecular docking study of phytochemicals from *T. riparia* against three target enzymes potentially important for the antibiofilm, anticancer and antiparasitic activity.

Sl. No.	Isolated phytochemicals from *T. riparia*	SaSrtA (PBD ID: 1T2W)	hCOX-2 (PDB ID: 5IKT)	LiTH (PDB ID: 2JK6)
1	Abieta-7,9 (11)-dien-13-β-ol	−6.5	−6.8	−6.7
2	Astragalin	−6.7	−8.4	−7.9
3	Boronolide	−5.5	−5.5	−6.2
4	Campesterol	−7.8	−7.6	−7.0
5	Deacetylboronolide	−5.1	−6.8	−7.0
6	Deacetylumuravumbolide	−5.3	−6.7	−6.6
7	Dronabinol	−6.6	−7.9	−7.0
8	Ibozol	−6.9	−7.3	−6.9
9	Luteolin	−7.5	−8.4	−7.6
10	Sitosterol	−7.6	−7.9	−7.1
11	Stigmasterol	−7.8	−8.0	−9.0
12	Tetradenolide	−5.2	−5.3	−7.0
13	Umuravumbolide	−5.8	−7.2	−7.2
14	1′,2′-Dideacetylboronolide	−5.9	−6.2	−5.9
15	6,7-Dehydroroyleanone	−6.9	−7.7	−7.2
16	7α-Hydroroyleanone	−6.9	−7.2	−6.6
17	8(14),15-Sandaracopima-radiene-7α,18-diol	−6.6	−7.1	−6.7
18	8(14), 15-Sandaracopima radiene-2α,18-diol	−5.8	−6.1	−5.7
19	9β,13β-Epoxy-7-abietene	−6.7	−7.4	−7.3
20	13-Epimanoyloxide	−6.6	−8.0	−7.2

SaSrt A: *Staphylococcus aureus* Sortase A, hCOX-2: Human cyclooxygenase-2, LiTH: *Leishmania infantum* trypanothione reductase.

## 7 Structure-Activity Relationship

**FIGURE 9:**
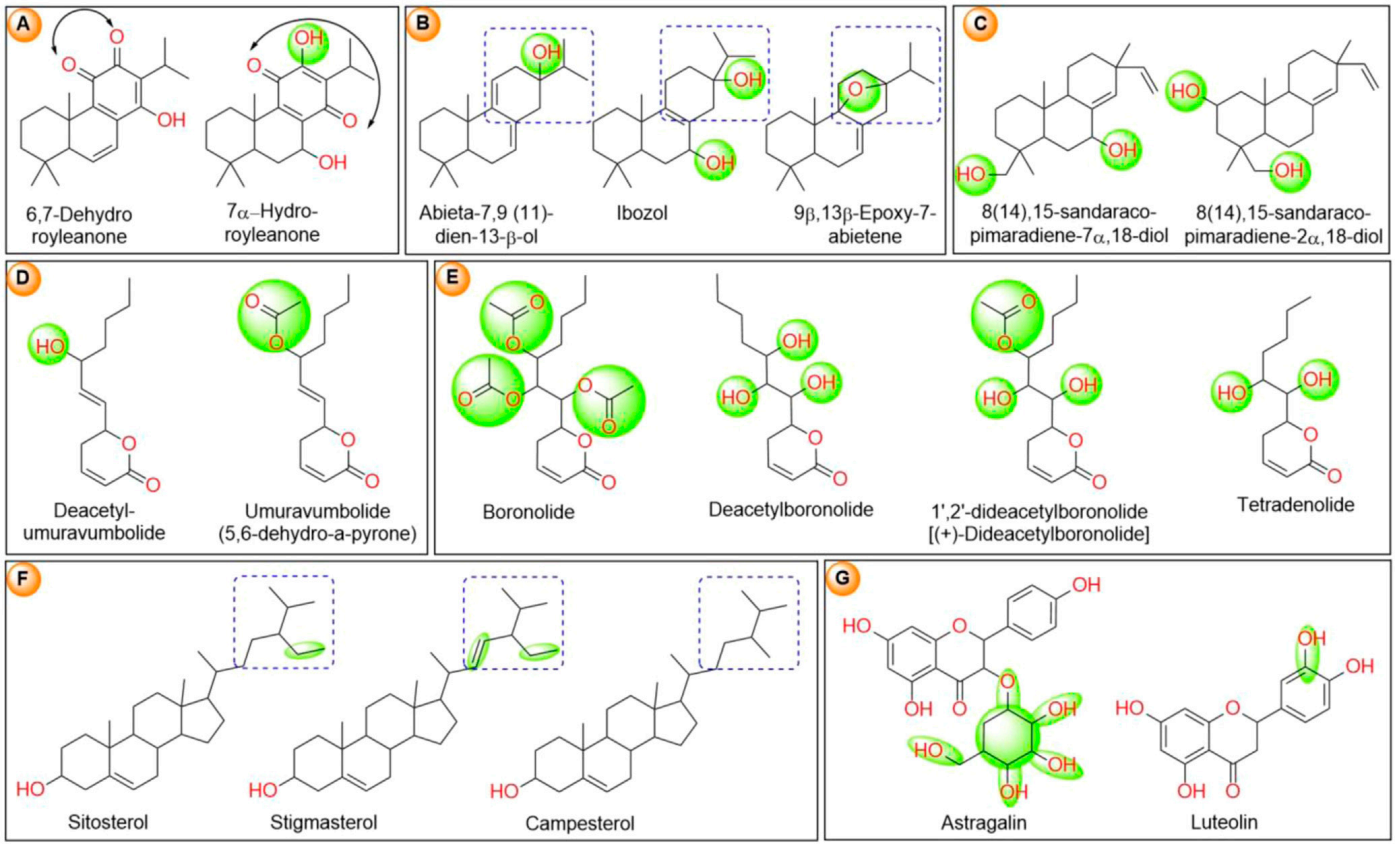
**(A–G)**. Structure-activity relationship analysis among phytochemicals reported from *T. riparia* with respect to biological activity in the form of generated docking score. The chemical structures are presented with the ChemDraw 18.0 software.

The structure-activity relationship (SAR) between each phytochemical in relation to its biological activity was analyzed mainly by the ChemDraw 18.0 software (Swain et al., 2021b). Three six-member fused ring royleanone diterpene derivatives, 6,7-dehydroroyleanone and 7α-hydroroyleanone have similar in structure, but the carbonyl (C=O) attachment at C-11 instead of C-13 in 7a-hydroroyleanone reduces the activity against LiTH more than for the other two targets (Figure 9A). Among three abietene derivatives, the one with a single hydroxy (-OH) groups (abieta-7,9 (11)-dien-13-β-ol) exhibited a comparatively lower docking score than the other two derivatives. At the same time the methoxymethane (CH_3_-O-CH_3_ or C_2_H_6_O) in 9β,13β-Epoxy-7-abietene was comparative potential to double hydroxy contained ibozol (Figure 9B) and overall, all are potential against hCOX-2. Similarly, methoxymethane with methyl or methoxy ethane (C_3_H_8_O) at the C-3 position in 8(14),15-sandaracopimaradiene-7α,18-diol showed reasonably higher biological activity than methoxymethane at same C-3 with additional -OH group at the C-1 position in 8(14), 15-sandaracopimaradiene-2α,18-diol (Figure 9C). Between two α-pyrone derivatives, the presence of hydroxy at the C-10 position in deacetylumuravumbolide exhibited a lesser docking score than methyl acetate (C_3_H_6_O_2_) attached umuravumbolide at the same C-10 position (Figure 9D). Overall due to the presence of -OH and C=O functional groups, they combined higher effectiveness against hCOX-2 other targets.

From another similar type of α-pyrone derivatives, boronolide, deacetylboronolide, 1′,2′-dideacetylboronolide and tetradenolide substituted by distinct -OH and C_3_H_6_O_2_ groups are not significantly influenced in biological activity; however, all four candidates were comparatively higher docking score against potential against LiTH (Figure 9E). From three steroid classes of constitutes, the presence of extra methyl group or 3-ethyl-4-methylpent-1-ene functional attachment in stigmasterol enhanced the biological activity mainly against LiTH than 3-ethyl-2-methyl pentane presented sitosterol and 2,3-dimethyl pentane presented campesterol (Figure 9F). Further, structural comparison between isolated two polyphenolic classes of compounds, astragalin (kaempferol-O-glucoside) and luteolin, the attachment of extract glucose at C-8 in astragalin showed similar activity with the extra -OH group at C-15 in the tetrahydroxyflavone moieties of luteolin (Figure 9G). However, the same astragalin has significantly lesser antibiofilm activity than luteolin. Thus, the position and substituted functional groups really influence the biological activity and the biological activity also varies by phytochemicals class (Swain et al., 2021a; Sahoo et al., 2021).

## 8 *In vivo* and Isolated Organ Studies

The methanolic extract from fruit, leaf, stem and root of *T. riparia* were tested at different concentrations on smooth (guinea pig ileum), skeletal (toad rectus abdominis), and uterine (non-pregnant guinea pig) muscle (Chagnon, 1984) . Only the leaf extract contracted the ileum (at 0.05 μg/ml). All extracts except that of the root contracted uterine muscle (at 500 μg/ml). Leaf extract contracted the skeletal muscle (at 50 μg/ml), whereas stem and fruit extract inhibited (both at 50 μg/ml) the contraction induced by acetylcholine (1 μg/ml), while root extract had no effect (up to 500 μg/ml). In the same study, hyper- and hypotensive effects of the extracts were tested *in vivo* in urethane-anesthetised rabbits, but no activity was observed from any extract at the dose used (5 mg/kg IV) (Chagnon, 1984). The diterpenediol from *T. riparia* has also been shown to possess papaverine-like antispasmodic activity on histamine, methacholine, and barium chloride-induced contractions of guinea pig ileum, as well as on noradrenaline-induced contractions of rabbit aorta (Van Puyvelde et al., 1987).

## 9 Toxicology

*T. riparia* taken in self-administered over-dosage of hot water extract as a remedy for cold or flu is reported in some cases of poisoning that occurred in adult males in South Africa during 18 years of clinical practice amongst Zulu communities. The symptoms included a severe toxic inflammatory response of mucous membranes, conspicuous at all body orifices, as well as profuse salivation. In more severe cases this went on to tissue necrosis and large-scale sloughing; In all cases of terminal illness, urine and stools consisted of almost pure blood; they were dark in color and contained shreds of exfoliated mucous membrane; The patients who were fatally ill went into anuria during the last 24–48 h, but one man recovered after 24 h of anuria (Bodenstein, 1977; Hutchings, 1996; Duke, 2002; Njau and Ndakidemi, 2017). No toxicity of methanolic leaf or stem extracts was observed upon intraperitoneal injection in mice at 1 g/kg (Chagnon, 1984). The cytotoxicity of essential oils and crude extracts from leaves, flower buds and stems of *T. riparia* was determined in Vero cells, with GI50 ranging from 143.00 ± 11.00 to 190.00 ± 15.00 μg/mL. As a positive control, ellipticin with GI50: 1.41 ± 0.06 μg/ml was used. The results indicated that the essential oil and the crude extract are non-toxic (unpublished data).

## 10 Conclusion

*T. riparia* is one of the most commonly used medicinal plants by indigenous communities of Africa. Commonly, it is planted close to homes to ward off mosquitoes and is traditionally used to treat several diseases including respiratory problems, cough, headache, stomach pain, diarrhea, fever, malaria and dengue etc. The active compound 8(14),15- sandaracopimaradiene-7α, 18-diol was isolated on several occasions through bioassay-guided purification, and has demonstrated multiple bioactivities, such as antispasmodic, anthelmintic and antimicrobial against mostly Gram-positive bacteria, including *M. tuberculosis* and *S. aureus* (both planktonic and biofilm). Other major bioactive compounds are 6,7-dehydroroyleanone (antimicrobial, antiparasitic), ibozol (antimicrobial, antitumor) and abieta-7,9(11)-dien-13-β-ol (antimicrobial), which was also documented in several experimental investigations. Moreover, essential oils demonstrated multiple bioactivities with major active compounds 6,7-dehydroroyleanone and 9β, 13β-epoxy-7-abietene. When all the isolated constituents were subjected to molecular docking using putative target enzymes: sitosterol, stigmasterol, luteolin and astragalin had the highest scores, but show little selectivity for any of the targets. This suggests that the have another mechanism of action. Based on *in vitro* evidence and calculated docking scores, 13-epimanoyloxide (−8 kcal/mol) against human cyclooxygenase-2 and 9β,13β-epoxy-7-abietene (−7.3 kcal/mol) against *Leishmania infantum* trypanothione reductase deserve further follow-up study *in vivo*. From our review it is clear that crude extracts have multiple *in vitro* effects, such as anticancer, analgesic, acaricide, insecticidal etc., but in most cases additional work is needed to isolate and characterize the active compounds. Further follow-up studies are also necessary to elucidate the mechanism of action and SAR. Also, pharmacokinetic and further toxicity studies will be required to assess their potential as drug candidates.

## Footnotes

1 Reproduced from “Endo EH, Costa GM, Nakamura TU, Nakamura CV, Dias Filho BP. Antidermatophytic activity of hydroalcoholic extracts from Rosmarinus officinalis and *Tetradenia riparia*. J Mycol Med. 2015 December; 25(4):274-279. doi: 10.1016/j.mycmed. 2015.09.003”. Copyright © 2015 Elsevier Masson SAS. All rights reserved.

2 Reproduced from “Demarchi IG, Thomazella MV, de Souza Terron M, Lopes L, Gazim ZC, Cortez DA, Donatti L, Aristides SM, Silveira TG, Lonardoni MV. Antileishmanial activity of essential oil and 6,7-dehydroroyleanone isolated from Tetradenia riparia. Exp Parasitol. 2015 October; 157:128-137. doi: 10.1016/j.exppara. 2015.06.014”. Copyright © 2015 Elsevier Inc. All rights reserved.

## Abbreviations

Me_2_CO, Acetone; ATCC, American Type Culture Collection (Manassas, Virginia, United States); C_6_H_6_, Benzene; BGF, Bioassay-guided fractionation; CHCl_3_, Chloroform; EtOAc, Ethyl acetate; FICI, Fractional Inhibitory Concentration Index; IC, Inhibitory concentration; IR, Infrared spectroscopy; MS, Mass spectral analysis; MeOH, Methanol; MIC, Minimal inhibitory concentration; MBC, Minimum bactericidal concentration; NMR, Nuclear magnetic resonance; SGCC, Silica gel column chromatography; TLC, Thin layer chromatography.
