# Supplementary material for: Ethnomedicinal, Phytochemical and Pharmacological Investigations of Tetradenia riparia (Hochst.) Codd (Lamiaceae)
Source: Front Pharmacol. 2022 Jun 2;13:896078. doi: 10.3389/fphar.2022.896078 (PMC9201335; doi:10.3389/fphar.2022.896078)
Supplement: Supplementary file 1 [file Table1.docx]

**Table S1:** Summary of bioactive compounds reported from various studies, including isolation and identification techniques.

| Extraction solvents | Purification steps | Identification techniques | Identified compound | Bioactivity | Remarks | Reference |
| --- | --- | --- | --- | --- | --- | --- |
| Acetone | Chromatography, CHCI_3_ and CHCI_3_ -Me_2_CO | Mass spectral analysis (MS); Nuclear magnetic resonance (NMR) | 7 α-hydroxyroyleanon, sitosterol, ibozol (1a) | - | Antitumor tests of ibozol against lymphocytic leukemia P388 indicated no activity up to doses of 200 mg/kg | (Zelnik et al., 1978) |
| Chloroform | Silica gel column chromatography (SGCC) | Infrared spectroscopy (IR), NMR | 8(14),15- sandaracopimaradiene-7α, 18-diol | Antispasmodic | - | (Van Puyvelde et al., 1982) |
| Chloroform | SGCC; n-hexane-toluene CHCl,-EtOAc-MeOH gradient; crystallization from cyclohexane | IR, NMR | 8(14),15- sandaracopimaradiene-2α, 18-diol | - | - | (Van Puyvelde et al., 1987) |
| Chloroform | SGCC (C6H6-CHC13-MeOH gradient), thin layer chromatography (TLC), crystallization from cyclohexane | IR, NMR | 1′,2′ - dideacetylboronolide | - | - | (Van Puyvelde et al., 1981) |
| Chloroform | SGCC (C6H6-CHC13-MeOH gradient), TLC | NMR, MS and IR | Sitosterol | - | - | (Van Puyvelde et al., 1981) |
| Chloroform | SGCC (C6H6-CHC13-MeOH gradient), TLC | NMR, MS and IR | Stigmasterol | - | - | (Van Puyvelde et al., 1981) |
| Chloroform | SGCC (C6H6-CHC13-MeOH gradient), TLC | NMR, MS and IR | Campesterol | - | - | (Van Puyvelde et al., 1981) |
| Methanol | SGCC, chloroform fractions eluted with C6H6/CHCl_3_, MeOH gradient | IR, NMR | Umuravumbolide, deacetyl-umuravumbolide and deacetylboronolide | - | - | (Van Puyvelde et al., 1979) |
| Methanol | SGCC | MS, NMR | 8(14),15- sandaracopimaradiene-7α, 18-diol | - | - | (Van Puyvelde et al., 1987a) |
| Chloroform | SGCC, C6H6/CHCl_3_, MeOH gradient | MS, NMR | 8(14),15- sandaracopimaradiene-7α, 18-diol | *Proteus vulgaris:* IC_50_= 25 μg/mL,  *Pseudomonas solanacearum:* IC_50_= 25 μg/mL,  *Shigella dysen teriae*: IC_50_= 12.5 μg/mL, *Bacillus subtilis:* IC_50_= 6.25 μg/mL,  *Mycobacterium smegmatis: Staphylococcus aureus, S. epidermidis, and Streptococcus pyogenes*: IC_50_= 12.5 μg/mL, *Candida al bicans:* IC_50_= 12.5 μg/mL, *Microsporum breuseghenii:* IC_50_= 25 μg/mL, *Microsporum gypseum:* IC_50_= 50 μg/mL | The compound has strong antimicrobial activity (MIC 6.25-12.5 µg/mL) against several Gram-positives, but the activity against Gram-negatives and mycelium fungi are selective. | (De Kimpe et al., 1982; Van Puyvelde et al., 1986) |
| n-hexane and CHCl_2_ | SGCC, EtOAc extract, hexane-EtOAc-MeOH gradient, crystallization | NMR | 5,6-dihydro-6-(1,2-dihydroxyhexyl)-2-pyrone | - | - | (Van Puyvelde and De Kimpe, 1998) |
| 70% (v/v) ethyl alcohol, maceration process | SGCC, hexane, hexane–dichloromethane -ethyl acetate-methanol gradient, TLC | NMR and gas chromatography (GC) | FR-I (abieta-7,9(11)-dien-13-β-ol), | *S. aureus*: IC_50_= 0.98 µg/mL, *Bacillus cereus* and *Enterococcus faecalis:* IC_50_= 31.2 μg/mL,  *Salmonella typhimurium:* IC_50_= 62 μg/mL, *E. coli:* IC_50_= 125 μg/mL | - | Fernandez et al. (2017) |
| 70% (v/v) ethyl alcohol, maceration process | SGCC, hexane, hexane–dichloromethane -ethyl acetate-methanol gradient, TLC | NMR and GC | FR-II (ibozol), | *S. aureus* and *E. faecalis:* IC_50_= 125 µg/mL, *B. cereus:* IC_50_= 62.5 μg/mL | - | Fernandez et al. (2017) |
| 70% (v/v) ethyl alcohol, maceration process | SGCC, hexane, hexane–dichloromethane -ethyl acetate-methanol gradient, TLC | NMR and GC | FR-III (8 (14), 15-sandaracopimaradiene-2α, 18-diol and 8 (14), 15-sandaracopimaradiene-7α, 18-diol) | *S. aureus:* IC_50_= 500 µg/mL, *E. faecalis:* IC_50_= 125 µg/mL, *B. cereus:* IC_50_= 250 μg/mL | - | Fernandez et al. (2017) |
| 70% (v/v) ethyl alcohol, maceration process | SGCC, hexane, hexane–dichloromethane -ethyl acetate-methanol gradient, TLC | NMR and GC | FR-IV (astragalin, boronolide and luteolin) | *S. aureus:* IC_50_= 500 µg/mL, *E. faecalis:* IC_50_= 250 µg/mL | - | Fernandez et al. (2017) |
| Hydrodistillation using Clevenger-type apparatus | SGCC, Pentane-dichloromethane-methanol gradient | MS, NMR | 6,7-dehydroroyleanone | *Leishmania amazonensis* promastigote (IC_50_=2.45 µg/mL; LD_50_ =16.9 µg/mL) after 24 h | Electron microscopy revealed that the oil is able to modify the promastigote ultrastructure, suggesting autophagy given chromatin condensation, blebbing, membranous profiles and nuclear fragmentation. | Demarchi et al., 2015 |
| Hydrodistillation using Clevenger-type apparatus | SGCC, Pentane-dichloromethane-methanol gradient | NMR | 6,7-dehydroroyleanone | Several species of *Mycobacterium tuberculosis* resistant to isoniazid, ethambutol, rifampicin; streptomycin, pyrazinamide and etionamide (IC_50_ =31.2 µg/mL) | The compound has selective activity against *Mycobacterium tuberculosis* while showing no strong effects on human microbiota. | Baldin et al., 2018 |
| Dichloromethane fraction | SGCC, bioassay guided fractionation (BGF); crystallization | MS, NMR | 8(14),15- sandaracopimaradiene-7α, 18-diol | *Caenorhabditis elegans,* IC_50_= 5.4 ± 0.9 µg/mL (17.8 ± 2.9 µM). | This compound is foremost responsible for anthelmintic activity. | Van Puyvelde et al. (2018) |
| Dichloromethane fraction | SGCC, BGF, crystallization | NMR | 8(14),15- sandaracopimaradiene-7α, 18-diol | *S. aureus:* IC_50_= 11.2 μg/mL,  *E. coli*: IC_50_= 209 μg/mL, *Shigella flexneri*: IC_50_= 212 μg/mL, *S. sonnei:* IC_50_= 210 μg/mL, *E. faecalis:* IC_50_= 16 μg/mL, *Listeria innocua:* IC_50_= 21 μg/mL, *Micrococcus luteus*: IC_50_= 16 μg/mL | This compound is bactericidal against *S. aureus* and has antibiofilm activity (BIC_50_ =8.8 ± 1.5 μg/mL) while more effective in planktonic cells (11.4 ± 2.8 μg/mL). | Van Puyvelde et al. (2021) |
| Ethyl acetate fraction | SGCC; BGF; crystallization | NMR | Deacethylumuravumbolide | *E. coli*: IC_50_= 530 μg/mL, *S. flexneri:* IC_50_= 465 μg/mL, *S.* *aureus:* IC_50_= 638 μg/mL, *M. luteus*: IC_50_= 213 μg/mL | - | Van Puyvelde et al. (2021) |
| Ethyl acetate fraction | SGCC; BGF; crystallization | NMR | Umuravumbolide | *S. flexneri:* IC_50_= 253 μg/mL, *S.* *aureus:* IC_50_= 252 μg/mL, *E. faecalis:* IC_50_= 252 μg/mL, *Listeria innocua:* IC_50_= 521 μg/mL, *M. luteus*: IC_50_= 176 μg/mL | - | Van Puyvelde et al. (2021 |

Bioassay-guided fractionation (BGF); Infrared spectroscopy (IR), Mass spectral analysis (MS); Nuclear magnetic resonance (NMR)

Silica gel column chromatography (SGCC); Thin layer chromatography (TLC)
